# Automated acoustic detection of mouse scratching

**DOI:** 10.1371/journal.pone.0179662

**Published:** 2017-07-05

**Authors:** Peter Elliott, Max G’Sell, Lindsey M. Snyder, Sarah E. Ross, Valérie Ventura

**Affiliations:** 1 Department of Statistics, Carnegie Mellon University, Pittsburgh, PA, United States of America; 2 Department of Neurobiology, University of Pittsburgh, Pittsburgh, PA, United States of America; 3 Pittsburgh Center for Pain Research, University of Pittsburgh, Pittsburgh, PA, United States of America; 4 Department of Anesthesiology, University of Pittsburgh, Pittsburgh, PA, United States of America; 5 Center for the Neural Basis of Cognition, Pittsburgh, PA, United States of America; Oregon Health and Science University, UNITED STATES

## Abstract

Itch is an aversive somatic sense that elicits the desire to scratch. In animal models of itch, scratching behavior is frequently used as a proxy for itch, and this behavior is typically assessed through visual quantification. However, manual scoring of videos has numerous limitations, underscoring the need for an automated approach. Here, we propose a novel automated method for acoustic detection of mouse scratching. Using this approach, we show that chloroquine-induced scratching behavior in C57BL/6 mice can be quantified with reasonable accuracy (85% sensitivity, 75% positive predictive value). This report is the first method to apply supervised learning techniques to automate acoustic scratch detection.

## 1 Introduction

Chronic itch is major clinical problem that remains ill-addressed. There are hundreds of pathological conditions that result in chronic itch, such as atopic dermatitis, psoriasis, hepatic disease, renal disease, HIV and multiple sclerosis [1]. It is estimated that approximately 10% of the general population (and 30% of the elderly) suffer from itch, which negatively affects sleep, mood, and quality of life, thereby presenting an enormous health burden [1, 2].

Rodent models of acute or chronic itch are frequently used for both basic and preclinical research studies aimed at understanding itch and developing better therapies [3–6]. In these models, the scratching behavior is used as a proxy for itch. In response to pruritogens, mice and rats show stereotyped bouts of site-directed scratching. Scratch bouts are a highly conserved itch behavior that is characterized by a series of individual hindpaw-mediated scratches (typically three to six) in rapid succession, followed by licking of the hindpaw. Traditionally, this behavior has been quantified as an indirect measure of itch manually by recording behavior and scoring videos manually. However, this type of analysis is time consuming; thus, sample sizes are often fairly small, and large scale experimentation is infeasible. These issues underscored the need to develop an automated method for the detection of scratching that is inexpensive and scalable.

Several counting techniques have been proposed to address these issues. One strategy for automated counting is to place a metal ring around the mouse’s hind paws and track perturbations in an electromagnetic field in the cage [7, 8]. Others have used video analysis to track paw movement and detect scratching [9–11]. A third proposal was to discriminate movement measured by a force transducer under the mouse’s cage [12].

In this paper we propose a new method for automation through acoustic detection of scratches which is related to the template matching method of [13], applied to rodents, and [14], applied to humans. Acoustic detection is non-invasive and can be performed in the dark, which is important because mice are nocturnal and lighting conditions that enable the visual assessment of scratching are known to be stressful for mice. Additionally, audio recording does not require specialized equipment. Our method is a two-step procedure: the first step relies on a scan statistic to identify potential scratch bouts, and the second step uses a random forest classifier for supervised learning and final classification. Accuracy was assessed based on both random forest out-of-bag predictions and held out validation sets. We found that our method detects between 70% and 85% (depending on the recording) of the true scratches present with 75% predictive value (the proportion of claimed scratches that are accurate). Finally, we evaluated how much training data is needed for accurate classification and how well a trained classifier can extrapolate to a new recording.

## 2 Experimental setup

**Fig 1:**
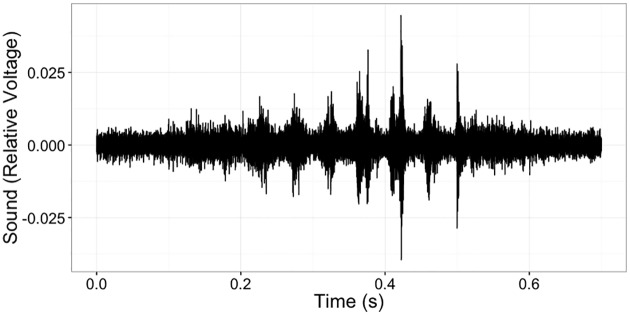
A 700 ms recorded sound wave including a scratch bout. The peaks correspond to individual scratches. This bout lasts approximately 400 ms.

Our data come from six recording sessions. In each session, researchers placed a lone C57BL/6 mouse in a sound proofed cage in a quiet room and injected chloroquine disulphate diluted in phosphate buffered saline (200 μg/20 μL) intradermally at the nape of the neck to induce itching. The amount of itch the mouse experiences is reflected in the frequency with which the mouse scratches itself. Our goal was to detect and measure this scratching automatically, rather than with manual observation. The raw audio data were samples from the soundwave across time (i.e., the *time domain*); an example time segment is shown in Fig 1. A video was used for manual identification of scratches, giving a ground truth with which we could measure accuracy. Specifications for the different recordings are given in Table 1. Note that the second recording was performed in a separate lab with different equipment from the other five recordings. We include this to gain some insight into how our method performs under varying conditions.

**Table 1:** Recording specifications.

Recording #	1	2	3	4	5	6
# Microphones	2	1	2	2	2	2
Audio Samp. Rate (kHz)	44.1	96	44.1	44.1	44.1	44.1
Bit Depth	16	16	16	16	16	16
Video Frame Rate (fps)	30	30	30	30	30	30
Time Recorded (min:sec)	35:15	8:23	45:16	45:28	46:15	46:23

**Fig 2:**
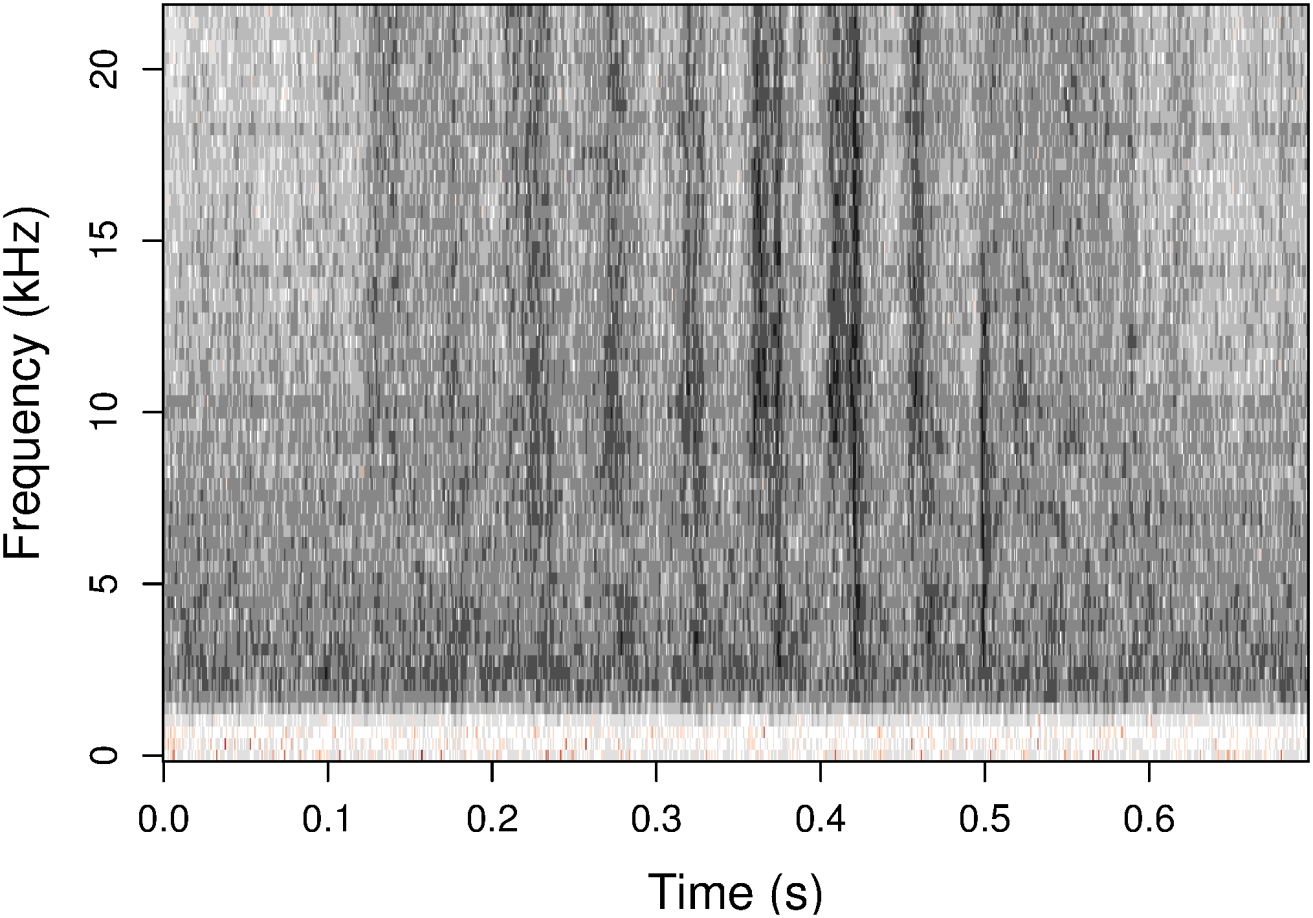
A spectrogram showing the short-time Fourier transform of the scratch bout from Fig 1. Vertical bands in the graph correspond to individual scratches. These bands are more distinct above 10 kHz.

Mouse scratching occurs in small sets of rapid swipes, which we refer to as *scratch bouts*. The goal of our procedure is to detect scratch bouts rather than individual scratches. The pattern of scratching can be seen in both the time domain and in a time-frequency analysis (e.g., a short-time Fourier transform), as in Fig 2. The time-frequency analysis decomposes the soundwave into audio pitch strength across time to give a richer representation of the signal.

Scratches within bouts tend to occur with a period of approximately 50 ms. A full scratch bout lasts between 200 and 500 ms but can be as long as a full second. Scratch bouts also tend to occur in groups, often with less than a second of separation, consistent with previous results [4, 15].

### 2.1 Ground truth labeling

We performed video-based labeling of scratch times by the naked eye. We first identified approximate scratch times with full-speed viewing, and then used frame-by-frame analysis and inspection of the audio spectrogram to more precisely label the onset and offset times of scratch bouts. As individual scratches are near in frequency to video frame rates (20 Hz vs. 33 Hz), there is occasionally some subjectivity in this method. Labels are precise up to approximately 50 ms, which is sufficient for the validation method described in Sec 3.3.

### 2.2 Ethics statement

This study was performed in strict accordance with the recommendations in the Guide for the Care and Use of Laboratory Animals of the National Institutes of Health. Animals were handled in compliance with an approved Institutional Animal Care and Use Committee (IACUC) protocol (#14043431) of the University of Pittsburgh. Animal suffering caused by the experiment was minimal.

## 3 Two-Pass method for scratch detection

Our proposed method has two main steps. First, we find time intervals where scratching may have occurred by applying a filter to the time-frequency representation of the audio signal and check for the rhythmic pattern that is characteristic of scratch bouts (Sec 3.1). Second, we use supervised learning to classify which of the selected intervals truly contain scratch bout (Sec 3.2); this requires a set of pre-labeled intervals to train the classifier and suitably chosen audio signal features that can distinguish between scratching and other confounding behaviors.

### 3.1 Identifying candidate islands

Our goal in the first step of the procedure is to pare down the full recording into a set of time intervals we call *candidate islands* that can be classified. This set should contain as many of the actual scratch bouts as possible; we are willing to tolerate collecting false positives as they can be removed during the classification step.

The collection process works by first identifying peaks in sound intensity that resemble individual scratches. We do this by applying a filter to a time-frequency representation of the recording then using a simple rule to automatically pick particular local maxima in the filtered signal. We are then able to aggregate peaks found across all microphones into groups matching the typical periodicity of scratches observed within bouts.

#### 3.1.1 Preliminary filtering

Scratch-like signals can be seen in the sound wave (Fig 1) but are not visible in all frequency bands (Fig 2). Filtering out noisy frequency bands will therefore yield a clearer signal. When an experiment involves more than one microphone, the filtering step operates separately on each microphone’s recording.

**Fig 3:**
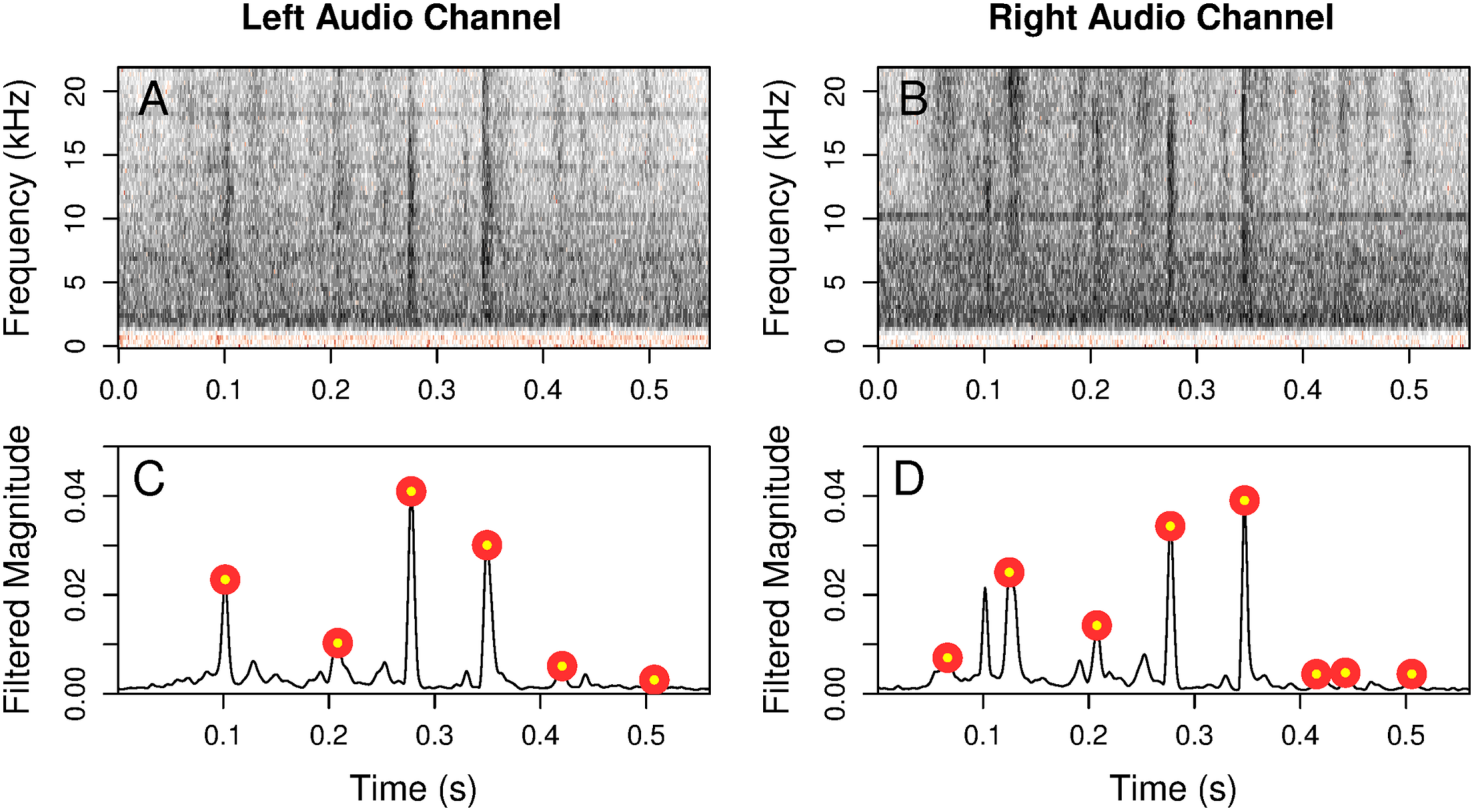
Spectrograms with the filtered signal. (A,B) Spectrograms of a scratch bout recorded on two channels. (C,D) Corresponding candidate islands. We observe variation between peak heights and number of peaks. The red dots indicate locations that were marked as peaks using the method described in Sec 3.1.2.

To get a time-frequency representation of the signal, we calculate a short-time Fourier transform using time bins of approximately 3 ms (128 samples at 44.1 kHz; 256 samples at 96 kHz), an overlap of three fourths of each bin, and a Gaussian window function. In each time bin, the modulus of each Fourier coefficient measures the strength of the signal at a given frequency, and this can be displayed in a spectrogram as in Fig 3A and 3B.

The power in the signal above 10 kHz is found by summing the squared Fourier coefficients corresponding to frequencies over 10 kHz. Doing this in each time bin gives us, for each microphone, a time series of power measurements. The time series is then smoothed using a triangular kernel smoother with a bandwidth of approximately 8 ms. An example result is shown in Fig 3C and 3D. The bandwidth was chosen to match the typical duration of an individual scratch. The triangular kernel was chosen to act approximately as a matched filter, with a triangle mimicking the expected change in power over the duration of an individual scratch.

#### 3.1.2 Peak detection and grouping into candidate islands

After obtaining the smoothed power measurements, we choose groups of local maxima that resemble scratch bouts: individual scratches should be short, sharp peaks in power, and scratch bouts should consist of groups of peaks with a period of approximately 50 ms.

**Fig 4:**
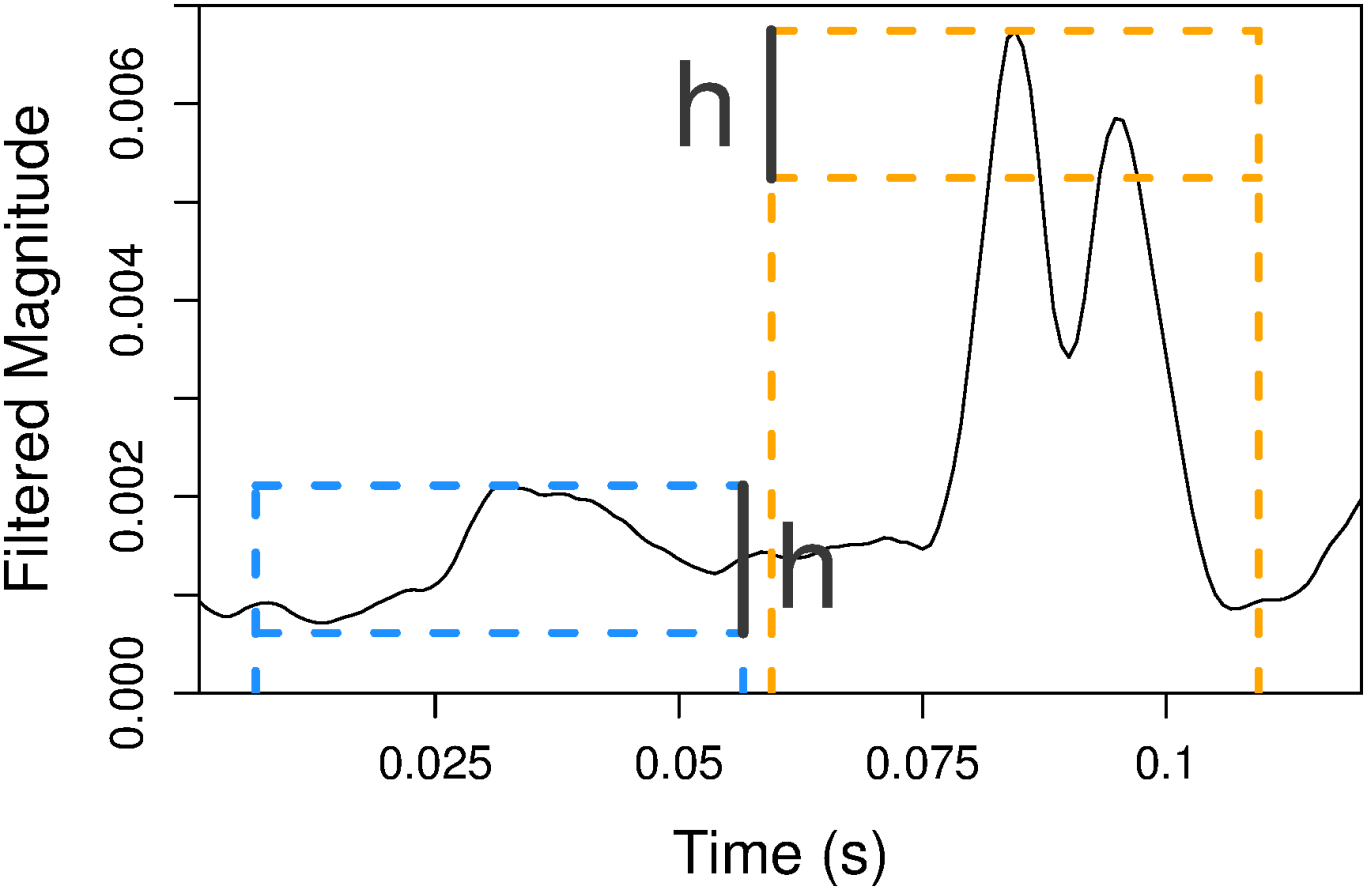
Two local maxima in the filtered signal. The top of each dashed box corresponds to the height of the local maximum, and the vertical dashed lines show the 25 ms band around the local maximum. The bottom of the dashed box shows the height of the local maximum minus the threshold *h*. On the right, the minimum in the interval is much smaller than the threshold, so the local maximum is considered a peak. On the left, the minimum in the interval is above the dashed line, so that local maximum is not considered a peak.

We define localized peaks in power to be points in the time series that are of maximal power within 25 ms and also exceed the minimum power in that interval by a chosen threshold *h*; see Fig 4. We can define this formally: if *P*_*t*_ is the smoothed power above 10 kHz at time *t* and $I_{t}^{25}$ is the time interval within 25 ms of *t*, then t* is a labelled peak time if it satisfies

$$\begin{array}{c} P_{t^{*}}=\max_{t\in I_{t^{*}}^{25}}P_{t},\;\text{and}\;P_{t^{*}}-h>\min_{t\in I_{t^{*}}^{25}}P_{t}. \end{array}$$ (1)

We can combine sets of peak times from two microphone channels by taking the union then merging any pair of points within 5 ms of each other. The remaining peaks are grouped into candidate islands by finding chains of peaks with less than 120 ms between adjacent peaks. Any chains with fewer than three peaks are discarded, yielding the final set of candidate islands. This procedure’s sensitivity to local maxima is controlled by the choice of *h*, which determines how distinct a peak in power must be from the surrounding signal. Because candidate islands will be checked again in the classification phase (Sec 3.2), we tune *h* to capture almost all scratch bouts, at the likely expense of including many candidate islands not corresponding to scratch bouts.

An example candidate island is shown in Fig 3. This example shows that the two microphones do not necessarily produce estimates of exactly the same peaks. There are also some local maxima in the time series of power measurements that are not significant enough to be considered peaks.

The template matching method [13] also uses acoustic recordings to detect scratches, but aims to count individual scratches instead of entire bouts. This is more ambitious because individual scratches have irregular waveforms even within bouts. The method first applies a bandpass filter to the signal and for each 50 ms interval, it calculates the log of the ratio of the sum of the squared time domain signal in the central 10 ms to the sum of the squared signal in the outer 40 ms. This template captures the idea that an individual scratch should cause a spike in the signal power lasting approximately 10 ms surrounded by periods of low power. Using this template gives a transformed time series analogous to Sec 3.1.1. Candidate scratches are taken to be peaks above a fixed threshold in this time series, and groups of candidate scratches with an approximate periodicity of 50 ms are labeled as scratches. This last step is analogous to Sec 3.1.2, although we use a local, not a fixed, threshold. Also, we do not constrain the peaks within a bout to be approximately equally spaced, so that candidate bouts that are contaminated by artifacts may be considered. Where the two methods differ more dramatically is that ours includes a random forest classification step. When we applied the method by Umeda et al. to our acoustic recordings we found that, in order to achieve 85% sensitivity to true scratches, there was a false discovery rate of 84%. This unacceptably high rate of false positives was likewise observed in our initial experiments when we used filtering but no classification. However, the classification step dramatically improves on this performance, resulting in an 85% sensitivity to true scratches with only a 25% false discovery rate. This increase in predictive value of automatic acoustic detection should thus enable reliable quantification of scratch behavior in mouse. We explain the classification step next.

### 3.2 Classifying candidate islands with random forests

After creating candidate islands, we use a trained classifier to get final labels. Labeling the candidate islands requires features that capture important characteristics of the data as well as a classification method capable of distinguishing between the observed features for scratches and non-scratches. Many of the features we use aim to characterize rhythmic patterns in the data. We also make use of audio pitch (frequency) information via the Fourier transform.

The feature extraction process gives us a high dimensional feature set, with many that are highly correlated. There are also potentially many complex interactions between features. The decision boundary for classification of scratches or non-scratch behavior is likely nonlinear. Random forest classifiers handle these particular properties well.

#### 3.2.1 Feature design

Most of our classifying features combine a transformation of the data, yielding a new time series of data, with a function extracting a characteristic of the transformed signal. Different transformations expose different patterns common among scratches, and the set of extraction functions provides a large body of informative features.

We first calculate a spectrogram, that is a short-time Fourier transform (Sec 3.1.1) and use it to obtain the six new time series described below:

1. We convolve the power above 10 kHz with a triangular kernel; this is the filter used for collecting candidate islands (Sec 3.1.1).
2. We convolve the power above 15 kHz with a triangular kernel.
3. We convolve the power above 20 kHz with a triangular kernel.
4. We first convolve the power above 10 kHz with a triangular kernel, then convolve the result with a Gaussian kernel.
5. We first convolve the power above 10 kHz with a triangular kernel, then convolve the result with a mixture of two Gaussian kernels.
6. At each time point we calculate a normalized inner product of the spectrum with an average of spectra drawn from a sample of scratches.

Then for each of these six new time series, we find peaks using the same technique as when collecting candidate islands (Sec 3.1.2), and we apply each of the following extraction functions:

1. The number of peaks.
2. The average time between peaks.
3. The standard deviation of times between peaks.
4. The mean, median, minimum, maximum, and standard deviation of the transformed time series.
5. The mean, median, minimum, maximum, and standard deviation of the transformed time series over the set of peaks.
6. The mean, median, minimum, maximum, and standard deviation of the full width at half maximum of each peak. The full width at half maximum captures the duration of a peak.

When an extraction function is not defined (e.g. if a time interval only has two peaks, then there is only one length of time between the peaks, so the third extraction function is not defined), the feature is coded as -1.

In addition to features extracted from time series transformations, we use features describing the frequency distribution across the candidate island. First we calculate a short-time Fourier transform using bins of length 50 ms and an overlap of 38.5 ms. Letting *c*(*t*, *ω*) be the Fourier coefficient for frequency *ω* at time *t*, we calculate a mean and maximum across time:

$$\begin{array}{ccc} c_{1}\left(\omega \right) & = & \frac{1}{T}\sum_{t=1}^{T}c\left(t,\omega \right), \end{array}$$ (2)

$$\begin{array}{ccc} c_{2}\left(\omega \right) & = & \max_{t\in \{1,\ldots ,T\}}c\left(t,\omega \right), \end{array}$$ (3)

where *T* is the number of time bins across the island, and

$$\begin{array}{c} m_{i,j,k}=\frac{\sum_{\omega }\omega^{k}c_{i}{\left(\omega \right)}^{j}}{\sum_{\omega }c_{i}{\left(\omega \right)}^{j}} \end{array}$$ (4)

for (*i*, *j*, *k*) ∈ {1, 2}^3^ to obtain the first and second moments of *c*_1_ and *c*_2_ with respect to *ω* for squared and non-squared coefficients.

Lastly, we include the duration of each candidate island as a feature.

#### 3.2.2 Improving accuracy with neighborhood-adjusted prediction

Given the numerous features for each candidate island, we could classify them as scratches or non-scratches independently of one another. However, we observed that scratch bouts do not occur independently but instead are clustered in time. That is, given a candidate island, if nearby islands are scratch bouts, it is more likely that the island of interest is also a scratch bout. Conversely, if nearby islands are not scratch bouts, it’s likely that the candidate island is a false positive as well. To take this into account we need an estimate of how likely scratch bouts are at any particular time. We estimate this likelihood from the random forest output as follows. Given a candidate island, we find all other candidate islands within 7.5 s. These neighbors are close enough that they’re likely to be part of the same behavior, be it scratching, walking, or grooming. We calculate the average predicted probability of scratching across the neighbors and multiply it with the original random forest predicted probability. Therefore, if there is an epoch where many candidate islands have high predicted probability, multiplying by the average probability will amplify the predicted probabilities for all candidate islands in that epoch, even ones that were previously classified as non-scratches.

### 3.3 Measuring prediction accuracy

To evaluate the usefulness of our method, we compare our predictions to the ground truth. The ground truth we use is a set of time intervals manually labeled from video as scratch bouts, and in general these intervals will not perfectly overlap with any candidate islands. Ideally there will be a single candidate island almost exactly matching a labeled scratch bout, but in some cases there is only (i) partial overlap, (ii) multiple candidate islands overlapping with a scratch bout, or (iii) multiple scratch bouts within a single candidate island. We process these various cases as follows. We count as a match any overlap of more than 50 ms between a scratch-classified candidate island and a manually labeled scratch bout, since this suggests that at least two swipes within the bout were correctly detected. Each one-to-one match between scratch bout and scratch-classified candidate island is considered a true positive. We also count one true positive for each scratch-classified candidate island that encompasses multiple manually labeled scratch bouts and each manually labeled scratch bout that encompasses multiple scratch-classified candidate islands. Any scratch-classified candidate island that fails to overlap with a scratch bout is considered a false positive.

Two common measures of classifier performance are the *sensitivity* and *false discovery rate*. The former is the proportion of actual scratch bouts we were able to find, that is

$$\begin{array}{c} \text{Sensitivity}=\frac{\#\text{True}\;\text{Positives}}{\#\text{Scratch}\;\text{Bouts}}. \end{array}$$ (5)

The false discovery rate (FDR) is the proportion of candidate islands we labeled as scratch bouts that actually weren’t, that is

$$\begin{array}{c} \text{FDR}=\frac{\#\text{False}\;\text{Positives}}{\#\text{True}\;\text{Positives}+\#\text{False}\;\text{Positives}}. \end{array}$$ (6)

A perfect classifier, with 100% sensitivity and 0% false discovery rate, is seldom achievable so we look for a classifier with the best tradeoff between the two rates. The tradeoff for a random forest is determined by the cutoff in predicted probability used for distinguishing between scratches and non-scratches. Increasing the cutoff raises the probability prediction necessary to consider a candidate island a scratch but decreases the sensitivity since fewer candidate islands will be deemed scratch bouts. This will also lead to fewer false selections, so as the cutoff increases the false discovery rate will tend to decrease.

To get accurate estimates of performance, we do not use the same data for both training and testing the classifier, because the classifier may overfit the training data leading to an optimistic performance estimate. Instead, we take advantage of the structure of the random forest to only measure predictions for observations not used in training. When training a random forest, each decision tree in the forest is trained on a random subset (a bag) of observations. We calculate the “out-of-bag” performance as follows. We measure the classification accuracy for a given candidate island by only counting votes from trees that were not trained on that candidate island. We repeat for each candidate island and count the number of true and false positive detections.

We cannot use out-of-bag predictions when testing the neighborhood-adjusted method (Sec 3.2.2). Indeed, any given candidate island is likely to be used to train decision trees used for out-of-bag predictions of its neighbors, so that the true classification unfairly informs the adjusted predicted probability, thus biasing the estimated accuracy. We evaluate the neighborhood-adjusted method by training and testing on separate recordings.

## 4 Results

We tested our scratch detection method on six audio recordings of mice with induced itching. Videoa of the experiment were also recorded for hand labeling of when scratching occurred. The manual labels were used as a ground truth against which our method could be compared. We measured accuracy using sensitivity, the proportion of true scratching that was successfully detected, and false discovery rate, the proportion of our predicted scratches that were incorrect (Sec 3.3).

**Fig 5:**
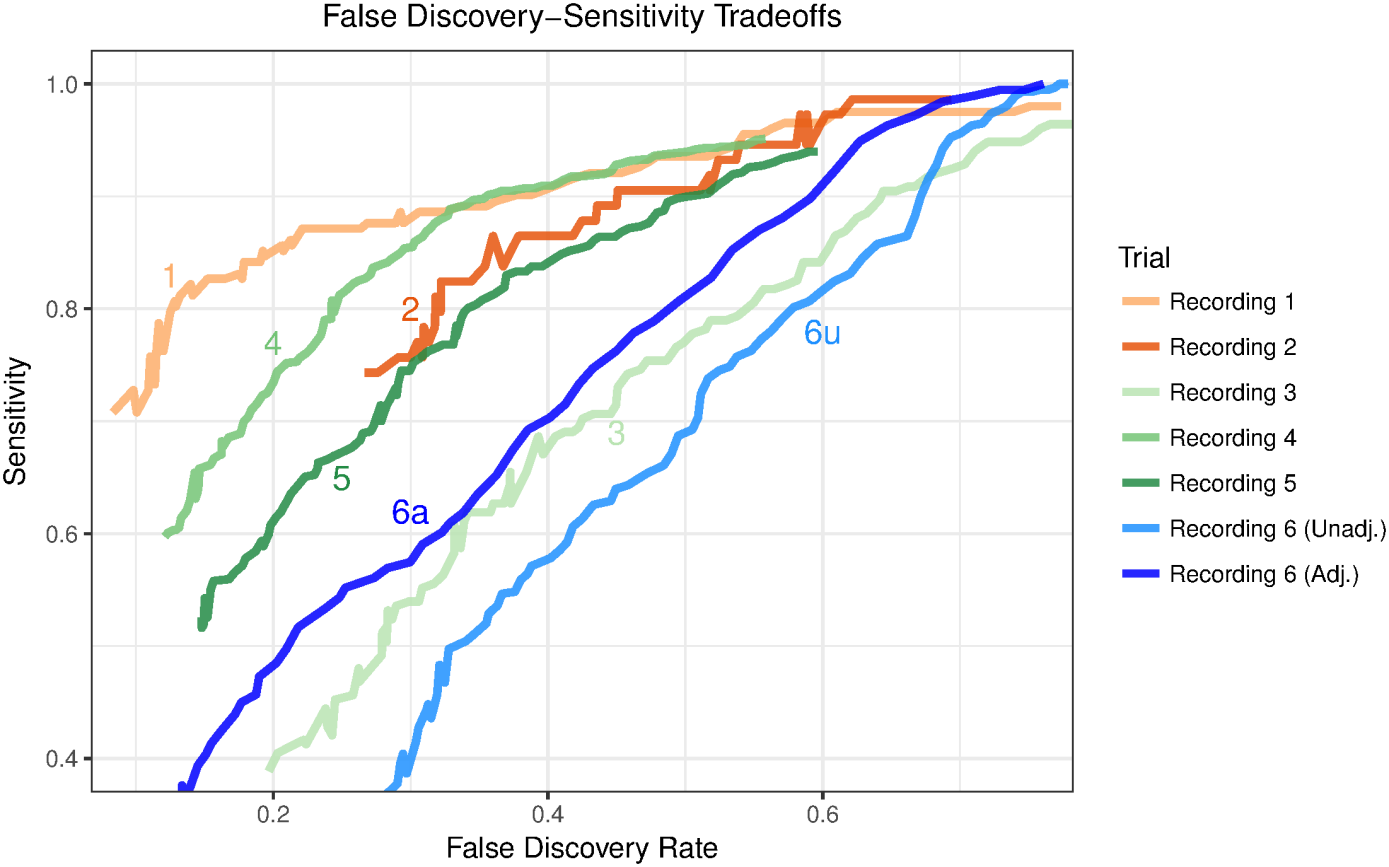
Classification accuracy for each recording. We compare the tradeoff between sensitivity and false discovery rate for out-of-bag predictions for the first 5 recordings. We also include results for recording 6 when using recording 5 to train the random forest. Performance is best for recordings 1, 2, 4 and 5. The neighborhood adjustment method (Sec 3.2.2) gives an improvement over the unadjusted cross prediction.

Fig 5 shows the performance of our method on all recordings. For recordings 1 to 5 we show the out-of-bag accuracy, and for recording 6 we show the accuracy of predicting scratch bouts with a classifier trained on recording 5, with and without neighborhood adjustment (Sec 3.2.2). We observed similar accuracy across recordings 1, 2, 4 and 5, with sensitivity ranging from 70% to 85% given a false discovery rate of 25%. The accuracy for recording 6 is much lower, at 55%, which suggests that it is better to use training data from a portion of the recording one wishes to automatically label rather than another data set. The neighborhood adjustment we applied to recording 6 gave a boost of about 10% in sensitivity for any given false discovery rate.

Table 2 summarizes the behavioral sources of false positive scratch detections in recording 1. The most common false positives were time periods where the mouse was walking around in its cage or grooming. It is likely that supplying labels in the training data for these confounding behaviors would improve performance.

**Table 2:** Sources of false positives in recording 1.

Behavior	Count	% of Labeled Scratches
Walking Around	15	8.1%
Grooming	15	8.1%
Standing on Hind Legs	1	0.1%
Sitting Down	3	1.6%

**Fig 6:**
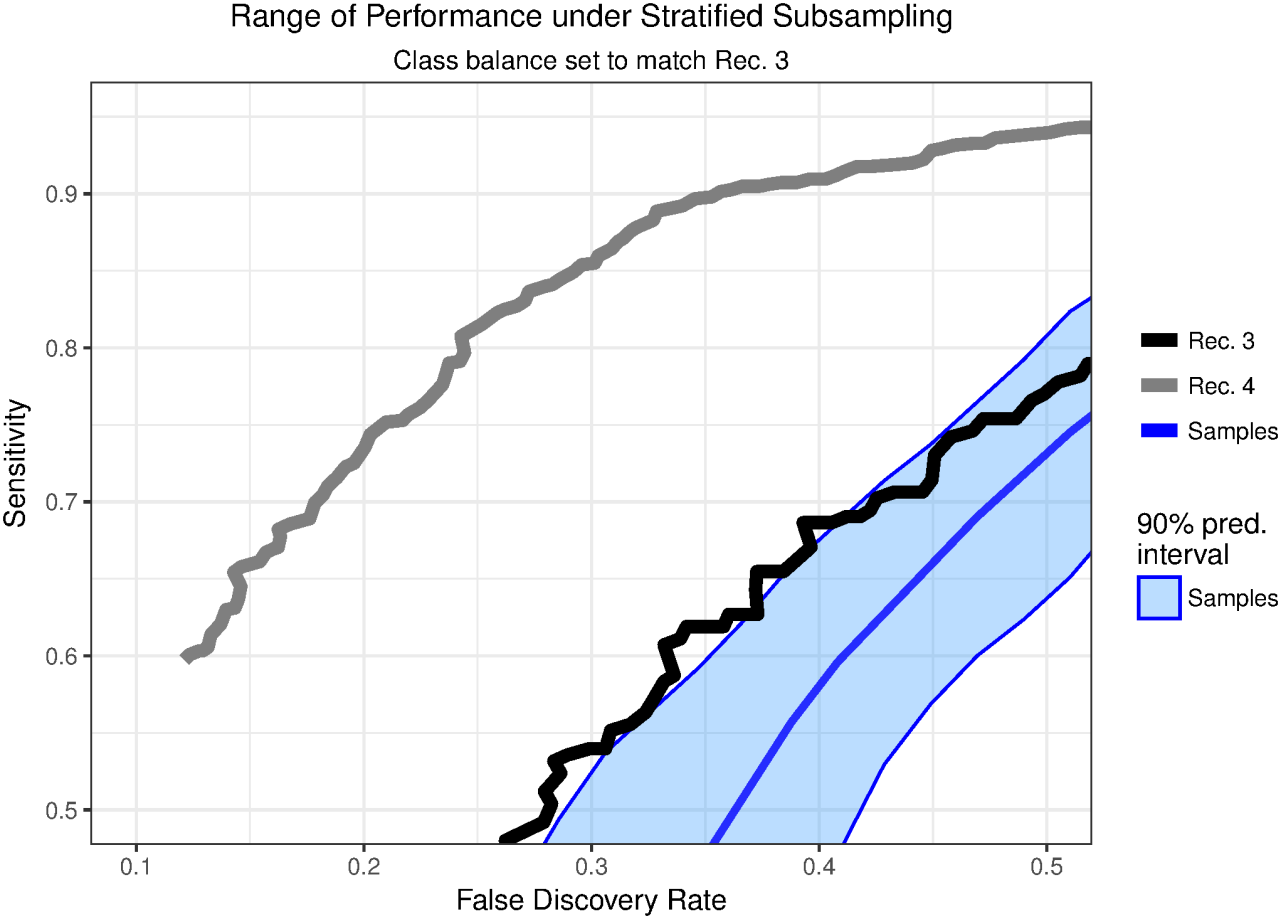
Performance response to class balance. We compare the results from recordings 3 and 4 to results for stratified subsamples of candidate islands from recording 4. The subsamples draw separately from true scratch bouts and false candidate islands to match the class balance for recording 3. The tradeoffs for recordings 3 and 4 are plotted alongside an interval covering 90% of 30 stratified subsamples from recording 4. The performance under subsampling is comparable to that of recording 3.

Fig 5 also shows that the classifier’s performance is inferior for recording 3. This happened because there is only a small number of scratches in the recording. We show this by drawing stratified subsamples from recording 4 to simulate recordings with varying amounts of true scratches. In recording 3, we found 2,090 candidate islands, with 255 (12%) true scratch bouts, compared to 2,880 candidate islands and 810 (28%) true scratch bouts in recording 4. We sampled separately from true scratch bouts and false candidate islands in recording 4 to simulate the lower scratching rate in recording 3. Fig 6 shows the tradeoffs between sensitivity and false discovery rate for recordings 3 and 4 together with a 90% simulation interval of this tradeoff in 30 stratified subsamples from recording 4. The results under stratified subsampling are comparable to the results for recording 3. This shows that it is more difficult to adequately detect scratching when it is less common.

**Fig 7:**
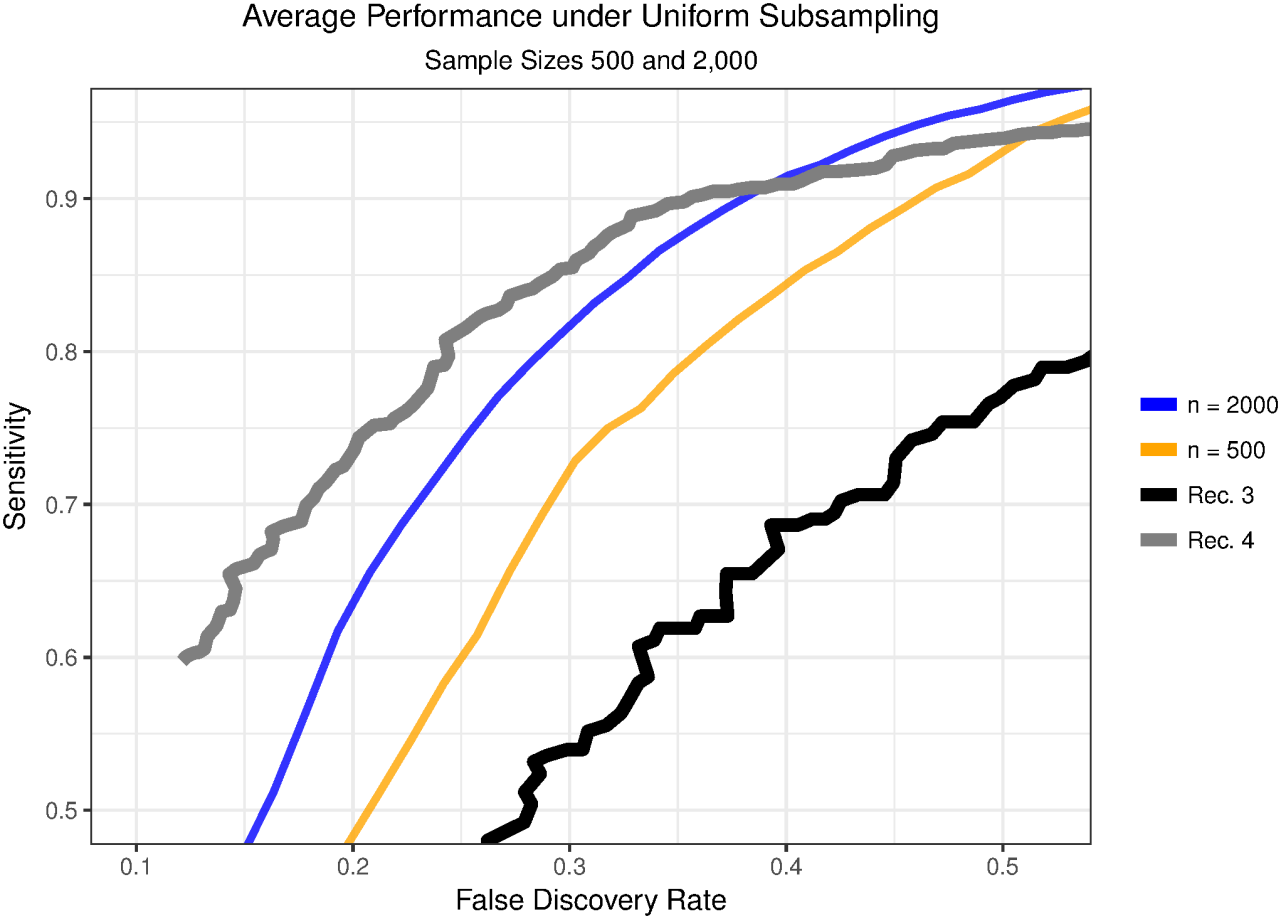
Performance response to training sample size. We compare the results from recordings 3 and 4 to results for subsamples of candidate islands from recording 4. The FDR-sensitivity tradeoffs for recordings 3 and 4 are plotted alongside the average tradeoffs across 10 subsamples from recording 4 of size 2,000 and 500.

Our algorithm classifies automatically but one choice we must make is the amount of labeled data to use when training the classifier. Fig 7 shows the tradeoff between sensitivity and false discovery rate for recordings 3 and 4 along with the average tradeoff for sets of 10 uniform subsamples of size 500 and 2,000 from recording 4. When using subsamples of size 2,000, we lose on average between 5 and 10% sensitivity, depending on the false discovery rate. For subsamples of size 500, we lose on average between 15 and 20% sensitivity.

**Fig 8:**
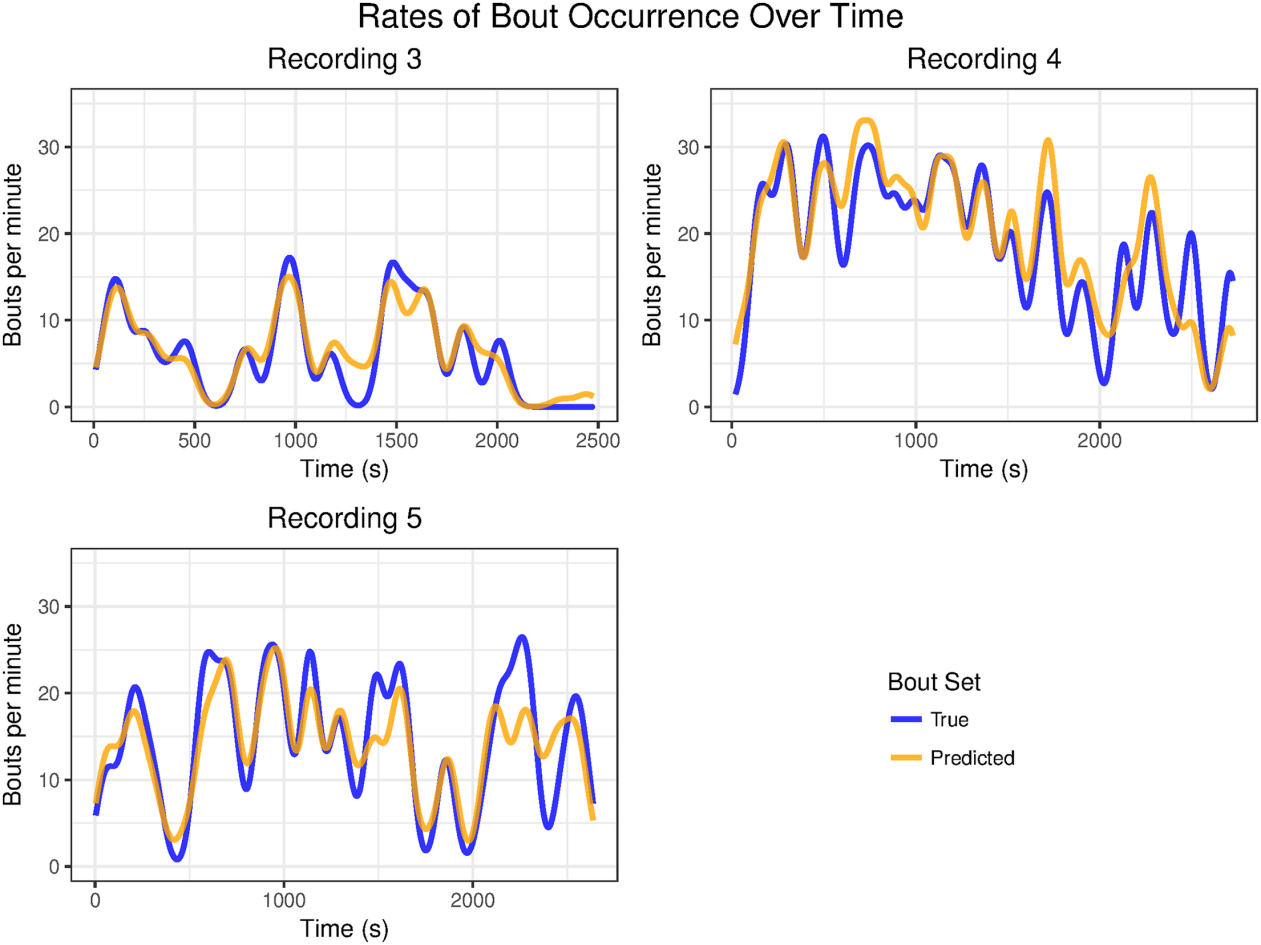
Actual vs predicted scratching rates over time. We compare the true rates of scratching over time estimated using kernel smoothing to rates based on out-of-bag predictions from our method. We see that important trends are picked up, e.g. the decline in scratching over time in recording 4 and the three large groups of scratching in recording 3.

Our method also allows us to estimate changes in itch over time. Fig 8 compares the rates of out-of-bag predicted scratch bouts to the true rates of scratch bouts in recordings 3, 4 and 5. The rates at each time point are estimated using kernel smoothing. We see that the fluctuations in scratching over time are closely matched by the fluctuations in predicted scratch rates. The ability to analyze itch behavior over time may be particularly important to study conditions of chronic itch. Most pathological conditions of pruritus result in persistent itch lasting days, rather than minutes, which is much more difficult to quantify manually. In contrast, the automated detection of scratching behavior would greatly facilitate the analysis of itch over extended time periods.

## 5 Conclusion

In this paper, we proposed a method for acoustic detection of mouse scratching behavior. Our method uses a scanning statistic to detect time intervals where scratching may have occurred then supervised classification via random forest and adaptive thresholding to generate final predictions. Applying this supervised approach to many descriptive statistics from potential scratch bouts allowed us to use more complex rules to discriminate between scratching and similar activities than any possible hand-designed rule, leading to more accurate classification. We measured accuracy based on both random forest out-of-bag predictions and held out validation sets. We also evaluated how much training data is needed for good classification and how well a trained classifier can extrapolate to a new recording. We find that our method detects between 70% and 85% of the true scratches present with only 25% of claimed scratch bouts erroneous. We show that performance depends on the amount of scratching in the recording and improves when training and predicting on the same recording. Thus, we recommend using labeled data from a portion of the same recording to get best results, as well as labeling other potentially confounding behavior. Overall, this method enables the quantification of itch using simple equipment and automatic detection with reasonable accuracy.

Traditionally, animal behaviors such as scratching have been analyzed through careful human observation. However, there is recent interest in automated, large-scale analysis of behavior [16]. Although the accurate identification of complex behaviors remains a challenge, scratching is a highly stereotyped innate behavior, which may facilitate its identification by automated measures. One of the advantages of the method described here is that it is not a one-size-fits-all solution. Rather, the supervised approach allows for context-specific variation due to, for instance, differences in background noise, microphone placement, and other factors that will vary from one environment to another. As a result, though our results were collected from a single strain and small number of mice tested with a single pruritogen, it is likely that the approach will be broadly applicable to the audio detection of scratch. In this regard, it should be emphasized that the automated acoustic detection described here, while a significant improvement over previous automated methods [13], is still not as accurate as human observation, which remains the gold standard. Nevertheless, automated detection of itch remains an important goal. In this regard, it is noteworthy that acoustic method to quantify nocturnal scratching of atomic dermatitis patients using a wristwatch-type sound detector has recently been developed [14], and it is hoped that automated detection will provide an objective measure of itch that will be a useful endpoint in future clinical trials for pruritus.
